# Metabolomic Profiling from Formalin-Fixed, Paraffin-Embedded Tumor Tissue Using Targeted LC/MS/MS: Application in Sarcoma

**DOI:** 10.1371/journal.pone.0025357

**Published:** 2011-10-03

**Authors:** Andrew D. Kelly, Susanne B. Breitkopf, Min Yuan, Jeffrey Goldsmith, Dimitrios Spentzos, John M. Asara

**Affiliations:** 1 Division of Hematology/Oncology, Sarcoma Program, Beth Israel Deaconess Medical Center, Harvard Medical School, Boston, Massachusetts, United States of America; 2 Division of Signal Transduction, Beth Israel Deaconess Medical Center, Boston, Massachusetts, United States of America; 3 Department of Pathology, Beth Israel Deaconess Medical Center, Harvard Medical School, Boston, Massachusetts, United States of America; 4 Department of Medicine, Harvard Medical School, Boston, Massachusetts, United States of America; Baylor College of Medicine, United States of America

## Abstract

The relatively new field of onco-metabolomics attempts to identify relationships between various cancer phenotypes and global metabolite content. Previous metabolomics studies utilized either nuclear magnetic resonance spectroscopy or gas chromatography/mass spectrometry, and analyzed metabolites present in urine and serum. However, direct metabolomic assessment of tumor tissues is important for determining altered metabolism in cancers. In this respect, the ability to obtain reliable data from archival specimens is desirable and has not been reported to date. In this feasibility study, we demonstrate the analysis of polar metabolites extracted directly from ten formalin-fixed, paraffin-embedded (FFPE) specimens, including five soft tissue sarcomas and five paired normal samples. Using targeted liquid chromatography-tandem mass spectrometry (LC/MS/MS) via selected reaction monitoring (SRM), we detect an average of 106 metabolites across the samples with excellent reproducibility and correlation between different sections of the same specimen. Unsupervised hierarchical clustering and principal components analysis reliably recovers *a priori* known tumor and normal tissue phenotypes, and supervised analysis identifies candidate metabolic markers supported by the literature. In addition, we find that diverse biochemical processes are well-represented in the list of detected metabolites. Our study supports the notion that reliable and broadly informative metabolomic data may be acquired from FFPE soft tissue sarcoma specimens, a finding that is likely to be extended to other malignancies.

## Introduction

Metabolomics is the study of the metabolite repertoire with respect to different physiological environments, tissue types, or cells [1], [2]. Analogous to genomics, transcriptomics, or proteomics, it holds great promise in the quest for biomarker discovery, particularly in oncology since cancer cells harbor an altered state of metabolism according to the Warburg effect [3].

The low per-sample cost of performing LC/MS/MS in SRM mode, or nuclear magnetic resonance spectroscopy (NMR) makes metabolomic biomarker investigation particularly appealing [4]. The sensitivity and specificity of both technologies, particularly mass spectrometry have improved dramatically in recent years, leading to the publication of a number of studies, mostly profiling urine and serum specimens [4]. Although clinically useful information has come out of this work, one shortcoming of the field is a lack of research on tissue metabolites [5]. It has been shown that the tumor microenvironment places unusual stresses on cells and leads to a shift in cellular energy production and utilization. The rate of glycolysis has been shown to increase in tumors resulting in high lactate levels, and high concentrations of alanine and ammonium are also observed in various malignancies as a result of elevated glutamine degradation [6]–[8]. Therefore, there is reason to believe that studying the biochemical intermediates and end products within tumor tissue samples would enhance our understanding of cancer biology.

As in the other “omics” fields, metabolomics would greatly benefit from the ability to utilize formalin-fixed and paraffin-embedded (FFPE) tissue specimens acquired during routine medical care. Because of their widespread availability and long term stability, accurately profiling the metabolite content of these tumor samples could accelerate the rate of discovery of clinically useful metabolomic biomarkers.

While there is literature on LC/MS/MS based metabolomic profiling, to our knowledge, there have been no publications on the use FFPE tissue [1], [2], [9], [10]. In this pilot study, we examine the technical feasibility and reproducibility of using targeted LC/MS/MS to profile FFPE specimens. We examine the degree to which malignant and non-malignant tissues can be distinguished by metabolite content, and we comment on the diversity of biochemical processes than can be probed with this method. The results of this work demonstrate that metabolomic studies of archival cancer specimens may yield reliable data and could aid investigators in identifying clinically and biologically meaningful biomarkers.

## Results

### Study Work Flow

**Figure 1:**
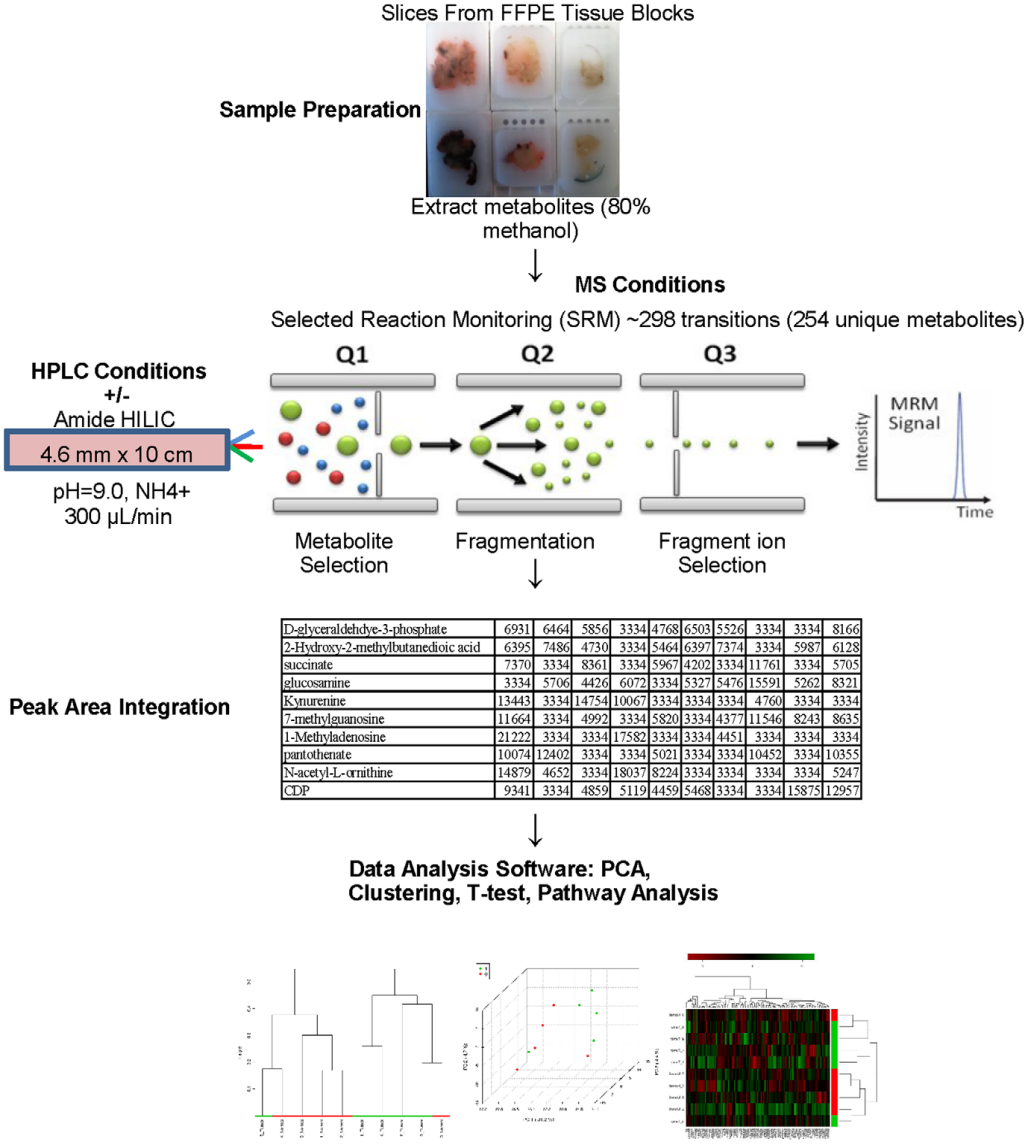
Flow chart summarizing targeted LC/MS/MS data acquisition.

A flow chart in Figure 1 summarizes the LC/MS/MS protocol with a complete methods description presented in the materials and methods section. We profiled a set of five formalin-fixed, paraffin-embedded soft tissue sarcoma specimens and five paired normal tissue samples by LC/MS/MS. Details on the specimen age and tissue type are presented in Table 1. All paraffin blocks contain tissue derived from surgical resection procedures. The specimens are all dated from 2004, 2005, or 2006 and one patient received radiation and chemotherapy prior to surgery. As this study aims to demonstrate feasibility and reliability of data acquisition, clinical variables aside from tissue type were not considered. To assess the biological reproducibility we ran LC/MS/MS on each of two independent sections from every paraffin block. In addition, for every section, we performed three independent injections to determine assay reproducibility.

**Table 1:** Characteristics of specimen cohort.

Sample	Year	Normal Tissue Type	Tumor Type
1	2005	Fibroadipose tissue	High grade sarcoma, myogenic differentiation
2	2005	Vein	High grade leiomyosarcoma
3	2006	Fibroadipose tissue	Monophasic synovial sarcoma
4	2005	Skeletal muscle	Biphasic synovial sarcoma
5	2004	Skin and subcutaneous adipose tissue	Well-differentiated liposarcoma

Information about the tissue type of each sample pair is provided. The year column refers to the year of surgical resection.

### Metabolite Detection

Upon acquiring raw data, we observed that out of the 249 compounds screened for by SRM, the number of unique metabolites robustly detected ranged from 74 to 143 across the samples with a mean value of 106 and represented several metabolic pathways. Interestingly, there is a trend towards higher metabolite detection rates in the tumor samples. Compared to results from unrelated experiments with fresh frozen tissue using the same platform, these numbers are approximately 40% lower, which is expected when analyzing harshly treated and aged specimens. Because frozen tissue from the patients in our study does not exist, we cannot make a direct comparison with FFPE data, however, in fresh tumor tissue we can routinely detect nearly 200 metabolites with robust signal across central pathways, including amino acids. While the detection of amino acids was low in this study, those most frequently detected in our dataset are acidic and positively charged at physiological pH. The presence of arginine in fifty-five of the sixty runs further illustrates this point as one should expect the amino acid with the highest side chain pKa to be extremely soluble in methanol. We also detected glutamate and histidine in at least one run for six, and five of the ten samples respectively. Whether formal charge influences compound extraction is beyond the scope of this paper, but nevertheless these observations provide a tangible indication of the classes of compounds that may be retained or lost during our extraction protocol.

### Reproducibility Assessment

**Figure 2:**
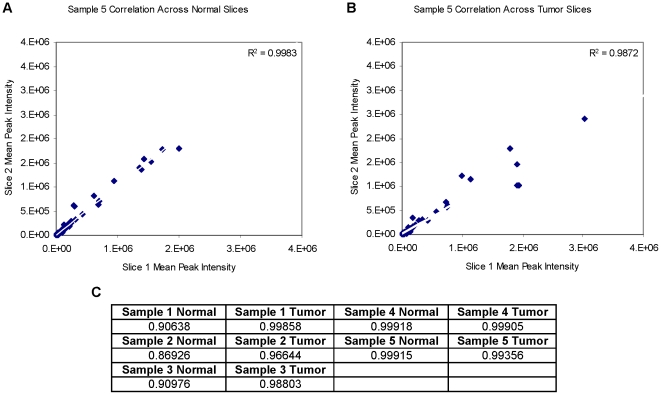
Technical performance summary. A, B) An example of the correlation between MS peak intensities from different sections from the same FFPE block is shown. C) Presentation of correlation coefficients for biological replicates. A full table with all correlation information is provided in the supplementary materials.

In order to assess the technical consistency across different injections into the mass spectrometer we calculated each pair-wise correlation coefficient across three injections for every FFPE section, excluding only those metabolites that were absent in all three sections. These values were consistently high for both normal and tumor tissue indicating that there is little variability between injections of the same sample preparation. The range of these correlation coefficients was 0.9373 to 0.9998 with very few under 0.9900 (Figure 2A, B). These complete data can be found in the Table S1. Next, we calculated the correlation coefficient between average peak intensities across the two sections from each FFPE block. Although lower than the values between injections, they still indicate a high degree of consistency across two sections from the same block with most correlations higher than 0.9000. Taken together, we can assert that LC/MS/MS data generated from FFPE tissue specimens are reproducible with respect to different injections of the same sample preparations and across different FFPE sections. A summary of the aforementioned analysis is presented in Figure 2C. It is important to note that in this part of our analysis no normalization or missing value imputation was performed so the concordances measured here reflect those of raw LC/MS/MS data.

### Differentiation between Known Phenotypes

After conducting the assessment of technical reproducibility, we excluded from further analysis any metabolites absent from ≥80% of the sixty LC/MS/MS experiments. The remaining missing values were then replaced by a value equal to half of the smallest measurement in the data, and we normalized as described in the methods section. Using normalized data, we examined the metabolite content across the tumor/normal pairs. Under the assumption that within the five sarcoma sub-types there would be a higher degree of metabolic deregulation, we calculated the variance for all detected metabolites across all samples with the hypothesis that the data would be more disperse in the tumors. Confirming our hypothesis, the variance in metabolite peak intensity across the tumor specimens was significantly higher than that of their normal counterparts (24.8% higher mean metabolite variance in tumors, P<0.05).

**Figure 3:**
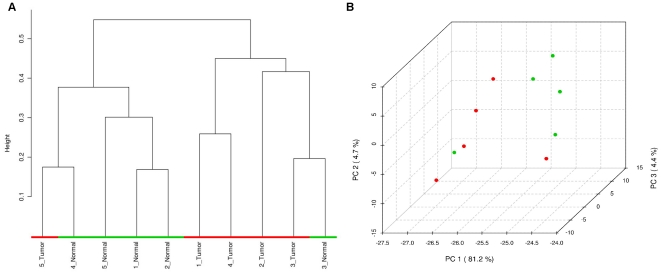
Unsupervised phenotypic distinction of samples. A) Hierarchical clustering and B) principal components analysis using data from the set of 119 metabolites passing filtering criteria. In the figures above, tumor specimens are in red and normal specimens are in green.

Having determined that the global metabolite content is more heterogeneous within our tumor cohort, we attempted to use unsupervised hierarchical clustering to investigate whether the sarcoma specimens would separate from their normal pairs. On the basis of 119 metabolites that passed the filtering criteria described above, we observed that the tumors clustered apart from the normal specimens with one exception. The dendrogram displayed in Figure 3A shows that one cluster contains four tumors and one normal specimen, while the other contains four normal tissue samples and one tumor. We hypothesize that the paired nature of our cohort may have prevented sample 3 and sample 5 from separating as the tumor and normal tissue samples from the same patient may be highly similar in metabolite content. Further examination of the pathology report revealed that the paraffin block containing normal tissue from patient 3 was contaminated with some tumor tissue and we reason that this may be why the pairing of samples from patient 3 was particularly difficult to break. The results of hierarchical clustering therefore suggest that a meaningful phenotypic distinction can be made on the basis of LC/MS/MS data from FFPE tissue.

As an additional method of unsupervised classification, we performed principal components analysis (PCA) on our cohort (Figure 3B). A similar phenomenon is observed as in the hierarchical clustering as the tumor and normal specimens separate in the first principal component with the exception of two samples.

### Exploration of Differentially Abundant Metabolites

We attempted to elucidate which metabolites may be most differentially abundant between our sarcoma specimens and their normal counterparts. To that end, we performed a student t-test and significance analysis of microarrays (SAM). These two methods yield mostly overlapping metabolites and they are presented in Table 2. Overall, we detected eight distinct differentially abundant metabolites between the two groups that illustrate the ability to detect differences in biochemical intermediates across FFPE tissue samples. Notwithstanding the small sample size, it is noteworthy that metabolites in this short list, such as cyclic-AMP, have already been shown to be altered in tumors.

**Table 2:** Differentially abundant metabolites identified by significance analysis of microarrays and by student t-test.

SAM Metabolite	D-value	P-value	T-Test Metabolite	P-value
Carbamoyl phosphate	3.1031	0.013193	cytosine	0.015548
CMP	2.8751	0.020588	Ng,NG-dimethyl-L-arginine	0.023861
ribose-phosphate	2.8668	0.020756	nicotinamide	0.027013
cytosine	2.7854	0.024622	cyclic-AMP	0.027947
cyclic-AMP	2.4982	0.037059		
DL-Pipecolic acid	2.301	0.04916		

**Figure 4:**
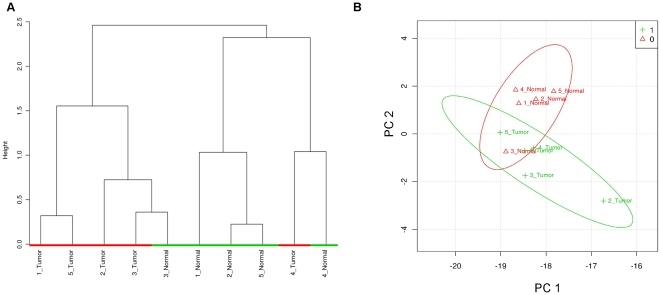
Supervised phenotypic distinction of samples. A) Hierarchical clustering and B) PCA of tumor and healthy specimens using data from the subset of significantly differentially abundant metabolites identified by t-test or SAM.

To confirm the relevance of this list, we performed supervised hierarchical clustering (Figure 4A) and PCA (Figure 4B) using only LC/MS/MS peak intensities for these metabolites, and indeed, we observe separation of the tumor/normal pairs.

### Diverse Biochemical Intermediates are preserved in FFPE Tissue

**Figure 5:**
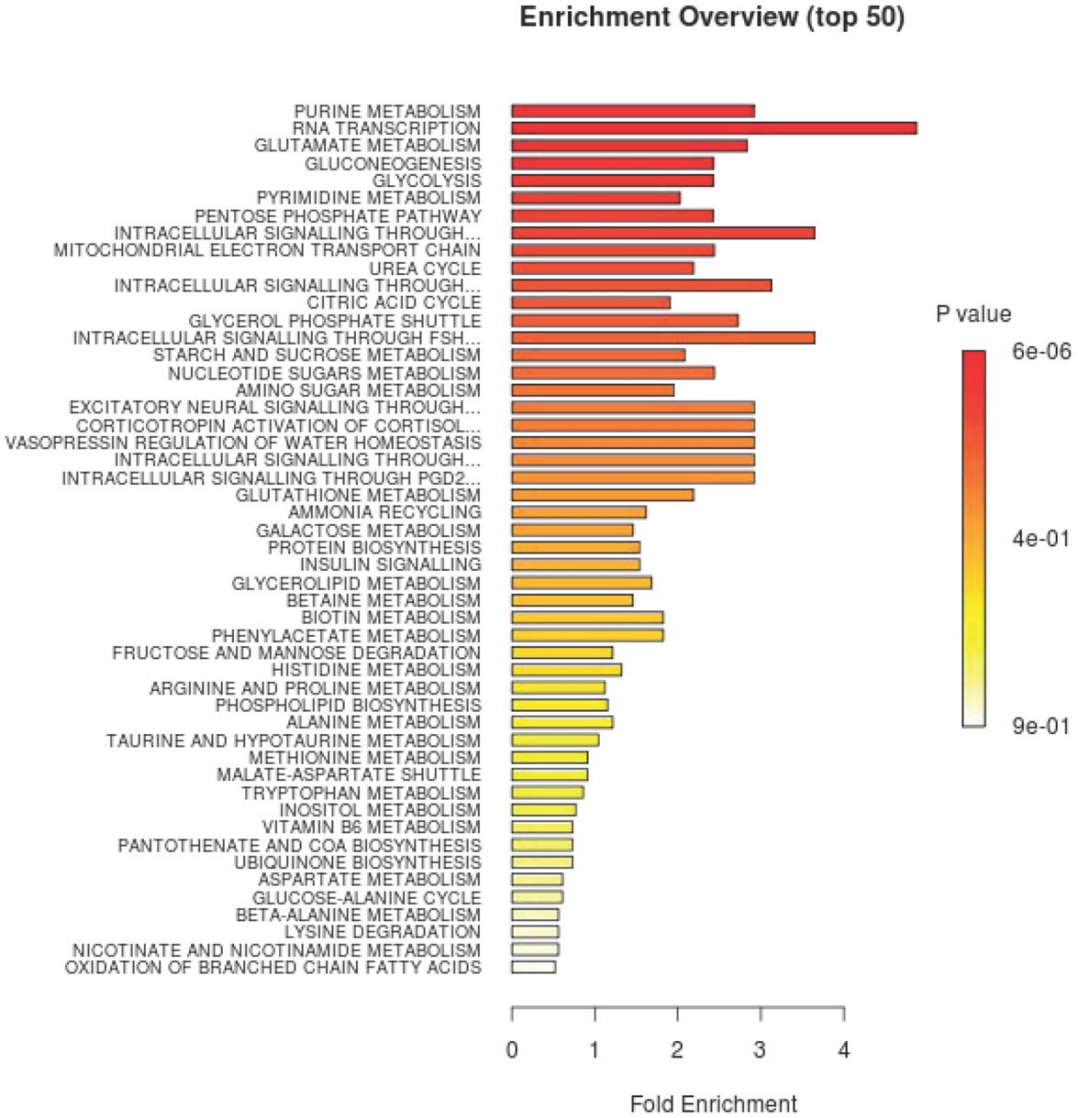
Summary of pathway enrichment analysis. Above is a display of the diversity of signaling pathways that are enriched on the basis of all 119 metabolites passing filtering criteria. The most significant p-values are in red while the least significant are in yellow and white.

Considering the treatment conditions of FFPE tissue, one could reasonably be concerned that metabolites recovered by LC/MS/MS may restrict analysis to relatively few pathways, with limited biological relevance. Thus, in order to confirm that the detected metabolites represent the preservation of a wide range of biochemical processes, we performed a pathway enrichment analysis on the entire list of 119 metabolites that passed filtering criteria (Figure S3). The enrichment analysis uses a list of compounds as an input and produces a list of known pathways which are most strongly represented by the input metabolites. Using our list, we identified a number of potentially impacted processes, many of which involve cellular energy production, although we see a wide range of metabolic pathways. This assures us that our detection of metabolites is not limited to a small subset of cellular processes, but rather encompasses a wide range of metabolic activities. It is interesting to note that several of the most significantly represented pathways - including glycolysis, glutamate metabolism, and the citric acid cycle - have been studied extensively in the context of cancer. In glycolysis, five out of eleven compounds are robustly detected; in glutamate metabolism, nine of eighteen are measured; and in the TCA cycle, five of eleven compounds appear in our data. A summary of this enrichment analysis is reported in Figure 5 and additional information regarding the specific metabolites detected from four highly represented pathways is presented in Figure S4 (4A – Glycolysis, 4B – Pentose Phosphate Pathway, 4C – Citric Acid Cycle, 4D – Glutamate Metabolism). In addition to the aforementioned analysis, we also ran the enrichment algorithm on our list of eight differentially present compounds and found that intracellular signaling processes involving cyclic-AMP were enriched along with several other pathways (see Figure S1). In contrast to the analysis performed on the full list of detected compounds, here we examined which pathways are likely to be altered in tumors compared to normal samples. To emphasize the key finding of these analyses, the pathways that are represented are diverse, which suggests that a broad spectrum of biochemical processes may be reliably interrogated using LC/MS/MS on FFPE specimens in future clinical studies.

## Discussion

Despite technological advances, there has been relatively little clinical metabolomics research performed on tumor tissue specimens [5]. And to our knowledge, there have been no publications demonstrating the successful acquisition of LC/MS/MS-based metabolomic data from FFPE tissue. In this study we show that reproducible data can be acquired from soft-tissue sarcoma sections preserved in paraffin blocks. Moreover, we demonstrate how these data can be used to probe a paraffin cohort for biochemical intermediates pertinent to phenotypic differences.

One concern with any LC/MS/MS-based metabolomic research protocol is the degree to which sample preparation degrades potential metabolites of interest [11], [12]. This challenge is amplified when one considers damage on the tissue during the process of formalin fixation and paraffin embedding. Limitations associated with FFPE tissue include degradation and attrition of compounds of interest. For instance, it has been shown that mRNA levels are significantly lower in FFPE preparations compared with frozen tissue extractions, and one may reasonably expect an analogous situation to arise in studying metabolites [13]. Therefore, a pipeline of obtaining reliable LC/MS/MS data from these challenging specimens would be a substantial step towards accelerating translational metabolomic studies. We observe a non-trivial degree of attrition of detectable compounds compared to the same protocol applied on both fresh and frozen tissue [14; unpublished data]. However, we still detect a substantial number of polar metabolites extracted from our FFPE cohort. Because frozen tissue samples from the patients in this study do not exist, we could not directly compare our data with those generated by high quality specimens, although this question is worth investigating in future work.

Other possible factors leading to compound attrition are degradation during formalin-fixation, loss of compounds during methanol extraction, and inefficient de-cross-linking of paraffin, although we cannot say to what degree these factors influence our detection rates.

Importantly, when we examine the raw data obtained from our protocol, we find that there is almost no variability between different injections from the same sample preparation. This assures us that the sample preparation protocol generates a homogeneous solution containing a wide range of metabolites. Furthermore, the tight correlation suggests that there is almost no technical variability across runs. Of particular importance to investigators though, is the high degree of correlation observed between different sections from the same paraffin block. For seven out of ten blocks, the correlation across sections was higher than 0.95, and for nine out of ten, it was above 0.90. These results suggest that measurements of metabolite content in paraffin embedded tissue may not be limited by a large sampling error.

A critical metric to assess the potential clinical research value of any “omics” application is the ability to detect known structure and information in the data. In addition, in the case of metabolomics one is interested in the breadth of metabolic pathways involved with the detected compounds. The ability to use unsupervised methods to accurately classify samples as being cancerous versus non-malignant is a very good indication of the value of this application and also suggests that at least a subset of compounds being detected are true indicators of differential metabolic activity. It is interesting to note that of the compounds identified as significantly altered, cyclic-AMP (detected by two different analytical methods) has been shown to be involved with several tumorigenic processes [15]–[17]. Moreover, pathways involved in cellular energetics – such as glycolysis, pentose phosphate metabolism, and the citric acid cycle – that have been implicated in cancer are significantly represented by the set of metabolites passing filtering criteria [6]–[8]. We also detected several phosphorylated metabolites that were identified as significantly altered, suggesting that phosphorylation may be better preserved in paraffin tissue than was previously believed. Taken together, these observations are consistent with the existing cancer literature and illustrate that many metabolic processes critical in cancer biology can be interrogated in FFPE tissue using LC/MS/MS.

To summarize, we have demonstrated the reproducible acquisition of metabolomic data using LC/MS/MS in SRM mode from FFPE soft tissue sarcoma specimens. Our data suggest that larger studies involving a more diverse cohort of malignancies may be warranted to further establish the reliability and general utility of the method, and that a wide range of clinical and biological questions may be successfully investigated with this methodology in widely available archived specimens.

## Materials and Methods

### Ethics Statement

This work was done in accordance with a protocol for archival tissue collection and use which was approved by the Institutional Review Board (IRB) at Beth Israel Deaconess Medical Center (BIDMC). The requirement for a patient consent form was waived by the IRB at BIDMC.

### FFPE Sarcoma Specimens

Formalin-fixed and paraffin-embedded tissue samples were retrieved by the Department of Pathology at Beth Israel Deaconess Medical Center. The tumor and normal tissue blocks were from the same patient and were obtained during surgical resection. For some specimens the tumor and normal tissue type is the same. It is important to note that for those samples that do not have an associated normal tissue of origin, e.g. synovial sarcoma, the normal tissue specimen was taken from areas directly adjacent to the tumor mass.

### Targeted Mass Spectrometry Analysis

From each of ten FFPE blocks, one 40 µm slice was discarded to minimize contamination of the specimens. The second and third 40 µm sections were then placed in two separate 1.5 mL microfuge tubes which correspond to the two sections analyzed from each sample. 1 mL 80% MeOH was added to each slice and mixed by vortexing. The sections were then incubated for 45 minutes at 70°C to melt the paraffin and extract metabolites. Next, the samples were placed on ice for 5 minutes, centrifuged in a cold room for 10 minutes at 14xG and the supernatant was transferred to a fresh 1.5 mL microfuge tube. The centrifugation was repeated once under the same conditions and the supernatant was again transferred to a new tube. The samples were dried by SpeedVac overnight. Samples were re-suspended using 35 µL HPLC grade water for mass spectrometry. 10 µL were injected and analyzed using a 5500 QTRAP triple quadrupole mass spectrometer (AB/Sciex) coupled to a Prominence UFLC HPLC system (Shimadzu) via selected reaction monitoring (SRM) of a total of 249 endogenous water soluble metabolites for steady-state analyses of samples. The 249 compounds monitored were chosen due to their involvement in central pathways important in a number of malignancies. Some metabolites were targeted in both positive and negative ion mode for a total of 298 SRM transitions. ESI voltage was +4900V in positive ion mode and –4500V in negative ion mode. The dwell time was 4 ms per SRM transition and the total cycle time was 1.89 seconds. Approximately 8–11 data points were acquired per detected metabolite. Samples were delivered to the MS via normal phase chromatography using a 4.6 mm i.d ×10 cm Amide XBridge HILIC column (Waters) at 300 µL/min. Gradients were run starting from 85% buffer B (HPLC grade acetonitrile) to 35% B from 0–3.5 minutes; 35% B to 2% B from 3.5–11.5 minutes; 2% B was held from 11.5–16.5 minutes; 2% B to 85% B from 16.5–17.5 minutes; 85% B was held for 7 minutes to re-equilibrate the column. Buffer A was comprised of 20 mM ammonium hydroxide/20 mM ammonium acetate (pH = 9.0) in 95:5 water:acetonitrile. Peak areas from the total ion current for each metabolite SRM transition were integrated using MultiQuant v2.0 software (AB/Sciex). The LC/MS/MS platform was quality controlled on a daily basis using polar metabolite extracts from H929 cancer cells. A selected number of metabolites were assessed using their known chromatographic elution times from the normal phase column and their expected peak area intensities.

### Technical Performance Assessment

Technical reproducibility was assessed across all pairs of injections of each section of each sample. There were sixty LC/MS/MS runs in all comprised of three 10 µL injections of each sample preparation. The Pearson correlation coefficient was taken between each pair of injections excluding only those metabolites for which none of the three injections registered a signal. The average peak intensities across all three injections were then taken for all metabolites and the Pearson correlation coefficient was computed across the average peak intensities for both sections from the same sample, excluding only those metabolites which had an average value of zero. The average number of metabolites detected was computed as a mean across all three injections for every section of every sample.

### Filtering and Normalization

The raw peak intensities were uploaded to Metaboanalyst.ca, an online freeware program intended for the analysis of metabolomic data [18], [19]. Metabolites were first removed from further analysis if they were absent from ≥80% of the 10 µL injections. We chose the cutoff of 80% in order to ensure that we did not exclude compounds that may be detected exclusively in one class (tumor or normal). Remaining missing values were calculated as half of the minimum positive value in the original LC/MS/MS output. The resultant data were normalized by sum and log2 transformed before advancing to statistical analysis. The resultant peak intensity matrix contained 119 unique metabolites which were used for further analysis. In some cases, LC/MS/MS cannot distinguish between isomers; we have intentionally omitted chemical side group position indices for these compounds. For completeness, we also attempted normalization by median followed by log2 transformation, which gave nearly identical results (see Figure S2A and B for hierarchical clustering and PCA respectively).

### Hierarchical Clustering and Principal Components Analysis

Unsupervised hierarchical clustering using the entire set of 119 metabolites passing filtering criteria was performed using the Pearson correlation metric and complete linkage. Principal components analysis was performed according to default settings on the metaboanalyst interface. Supervised clustering using the union of metabolites identified by t-test and SAM was done using the Pearson correlation metric and the Ward linkage method.

### Differentially Abundant Metabolite Identification

Differentially abundant metabolites were identified by a student t-test using a p-value cutoff of 0.05 and also by significance analysis of microarrays with a delta value of 0.4.

### Pathway Enrichment Analysis

Pathway enrichment (representation) analysis was performed using both the 119 compounds that passed our filtering criteria and using the union of compound lists identified by the student t-test and SAM. The metabolite set library used was the “metabolic pathway associated metabolite sets,” and representation analysis was done using the hypergeometric test. The output of this algorithm will mark a metabolic pathway as significantly represented by the input list of compounds if significantly more compounds involved with the pathway are present in the input list than would be expected by random chance. This analysis was implemented in Metaboanalyst.

## Supporting Information

Figure S1 **Pathway enrichment analysis summary using metabolites detected as significantly differentially present in tumor and healthy tissue.** Significant p-values are in red while less significant p-values are in yellow or white.
(TIF)

Figure S2 **Unsupervised phenotypic distinction of samples.** A) Hierarchical clustering and B) PCA of tumor and healthy tissue samples using data normalized by median.
(TIF)

Figure S3 **Table of pathways represented by 119 metabolites passing filtering criteria.** The column labeled “hits” presents the number of metabolites we detect from each pathway while the column labeled “total” gives the total number of intermediates in the process.
(TIF)

Figure S4 **Pathway representation summary.** The diagrams above indicate in boldface the metabolites we robustly detected which are involved in A) glycolysis, B) pentose phosphate pathway, C) citric acid cycle, and D) glutamate metabolism.
(TIF)

Table S1 **Complete summary of pair-wise correlations across injections for every section.** Included for every section are all pair-wise Pearson correlation coefficients (ρ) across three LC/MS/MS injections. The sections are denoted by sample number.section number_tumor/normal status; so the first slice from the tumor pair from sample five would be denoted: “Sample 5.1 Tumor.”
(DOC)
