# Supplementary figures and images for: Metabolomic Profiling from Formalin-Fixed, Paraffin-Embedded Tumor Tissue Using Targeted LC/MS/MS: Application in Sarcoma
Source: PLoS One. 2011 Oct 3;6(10):e25357. doi: 10.1371/journal.pone.0025357 (PMC3184969; doi:10.1371/journal.pone.0025357)

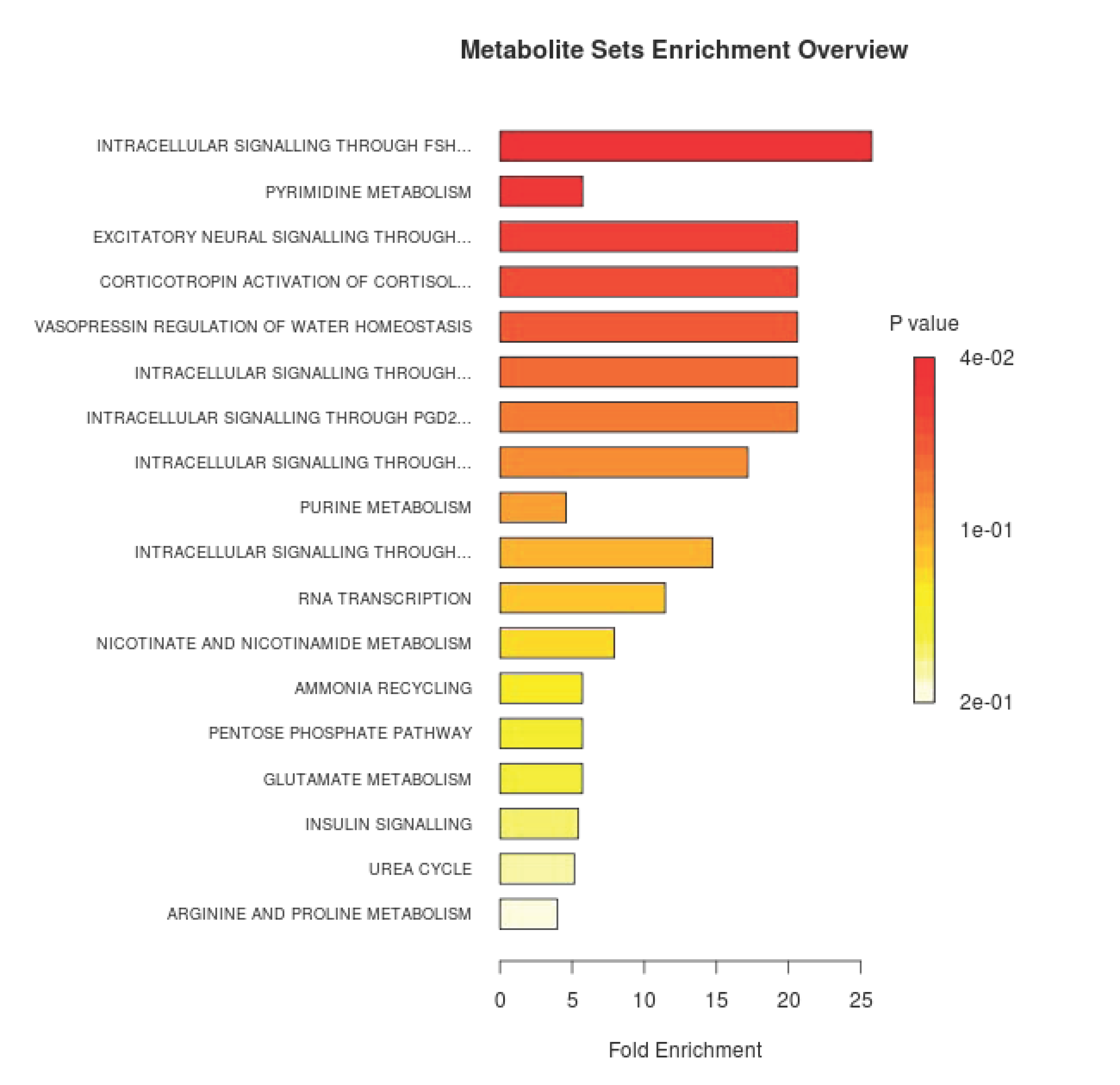

Supplement: Figure S1 — Pathway enrichment analysis summary using metabolites detected as significantly differentially present in tumor and healthy tissue. Significant p-values are in red while less significant p-values are in yellow or white. (TIF) [file pone.0025357.s001.tif]

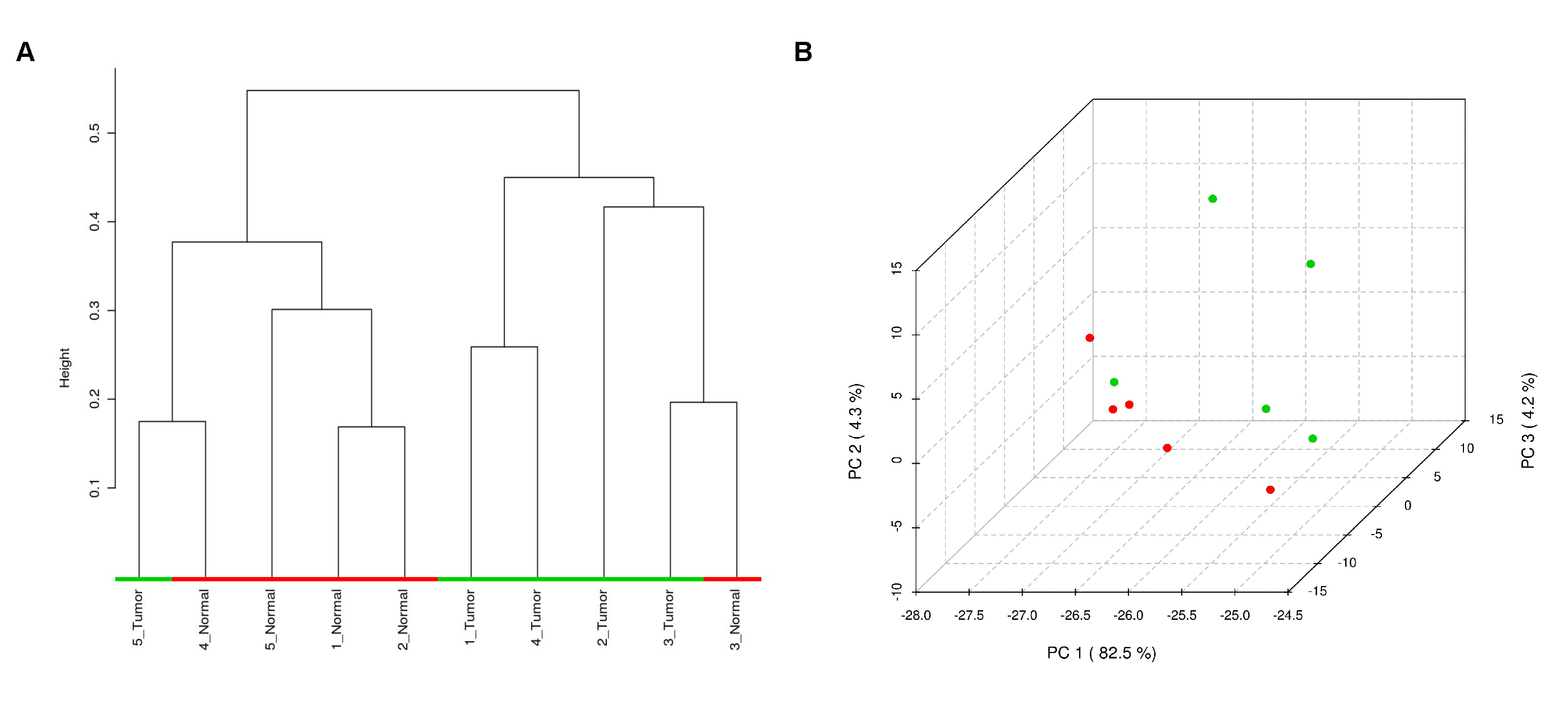

Supplement: Figure S2 — Unsupervised phenotypic distinction of samples. A) Hierarchical clustering and B) PCA of tumor and healthy tissue samples using data normalized by median. (TIF) [file pone.0025357.s002.tif]

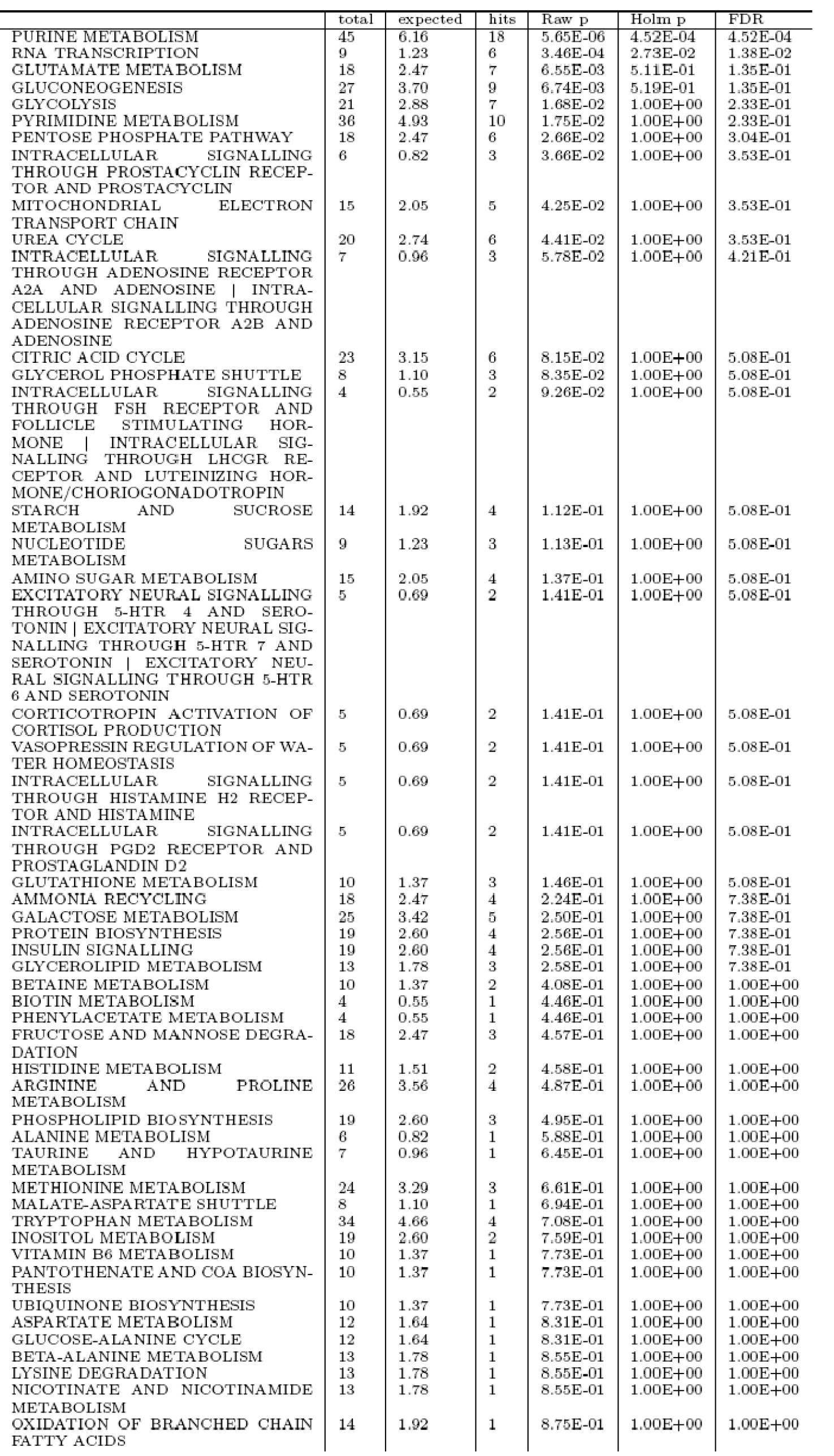

Supplement: Figure S3 — Table of pathways represented by 119 metabolites passing filtering criteria. The column labeled “hits” presents the number of metabolites we detect from each pathway while the column labeled “total” gives the total number of intermediates in the process. (TIF) [file pone.0025357.s003.tif]

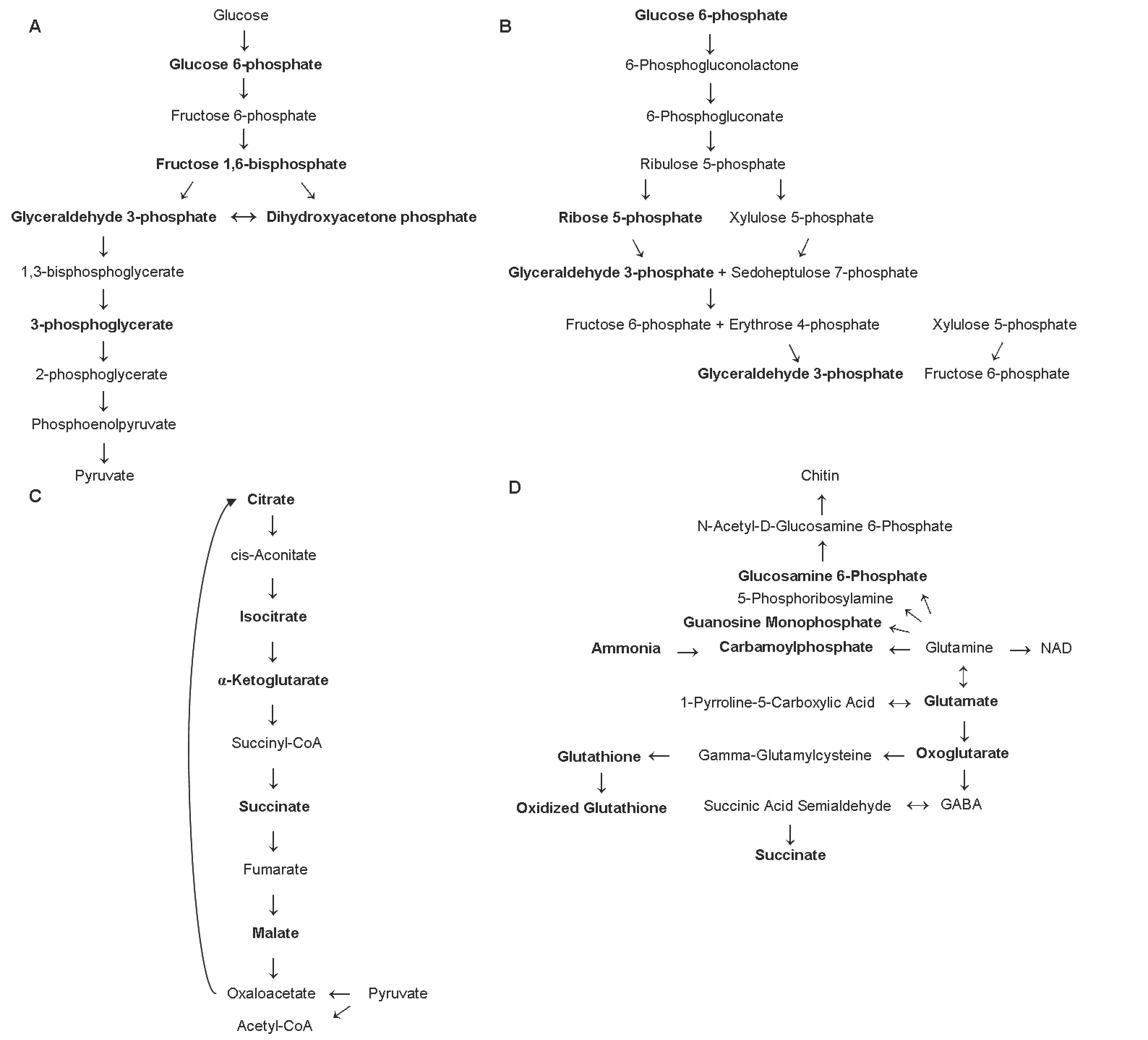

Supplement: Figure S4 — Pathway representation summary. The diagrams above indicate in boldface the metabolites we robustly detected which are involved in A) glycolysis, B) pentose phosphate pathway, C) citric acid cycle, and D) glutamate metabolism. (TIF) [file pone.0025357.s004.tif]
