# Supplementary material for: Metabolomic Profiling from Formalin-Fixed, Paraffin-Embedded Tumor Tissue Using Targeted LC/MS/MS: Application in Sarcoma
Source: PLoS One. 2011 Oct 3;6(10):e25357. doi: 10.1371/journal.pone.0025357 (PMC3184969; doi:10.1371/journal.pone.0025357)
Supplement: Table S1 — Complete summary of pair-wise correlations across injections for every section. Included for every section are all pair-wise Pearson correlation coefficients (ρ) across three LC/MS/MS injections. The sections are denoted by sample number.section number_tumor/normal status; so the first slice from the tumor pair from sample five would be denoted: “Sample 5.1 Tumor.” (DOC) [file pone.0025357.s005.doc]

| **Sample 1.1 Normal** | **Sample 1.2 Normal** | **Sample 1.1 Tumor** | **Sample 1.2 Tumor** | **Sample 4.1 Normal** | **Sample 4.2 Normal** | **Sample 4.1 Tumor** | **Sample 4.2 Tumor** |
| --- | --- | --- | --- | --- | --- | --- | --- |
| **ρ** | **ρ** | **ρ** | **ρ** | **ρ** | **ρ** | **ρ** | **ρ** |
| 1,2 | 1,2 | 1,2 | 1,2 | 1,2 | 1,2 | 1,2 | 1,2 |
| **0.96636** | **0.97313** | **0.99774** | **0.99745** | **0.99984** | **0.99862** | **0.99481** | **0.99822** |
| 2,3 | 2,3 | 2,3 | 2,3 | 2,3 | 2,3 | 2,3 | 2,3 |
| **0.98439** | **0.99556** | **0.99819** | **0.99788** | **0.99975** | **0.99921** | **0.99931** | **0.99894** |
| 1,3 | 1,3 | 1,3 | 1,3 | 1,3 | 1,3 | 1,3 | 1,3 |
| **0.97730** | **0.95901** | **0.99741** | **0.99446** | **0.99957** | **0.99964** | **0.99543** | **0.99749** |
| **Sample 2.1 Normal** | **Sample 2.2 Normal** | **Sample 2.1 Tumor** | **Sample 2.2 Tumor** | **Sample 5.1 Normal** | **Sample 5.2 Normal** | **Sample 5.1 Tumor** | **Sample 5.2 Tumor** |
| **ρ** | **ρ** | **ρ** | **ρ** | **ρ** | **ρ** | **ρ** | **ρ** |
| 1,2 | 1,2 | 1,2 | 1,2 | 1,2 | 1,2 | 1,2 | 1,2 |
| **0.99375** | **0.99436** | **0.98725** | **0.98677** | **0.99583** | **0.99839** | **0.99916** | **0.97719** |
| 2,3 | 2,3 | 2,3 | 2,3 | 2,3 | 2,3 | 2,3 | 2,3 |
| **0.99801** | **0.99835** | **0.99292** | **0.96387** | **0.99735** | **0.99803** | **0.99837** | **0.98457** |
| 1,3 | 1,3 | 1,3 | 1,3 | 1,3 | 1,3 | 1,3 | 1,3 |
| **0.99474** | **0.99716** | **0.99327** | **0.93733** | **0.99895** | **0.99945** | **0.99916** | **0.99840** |
| **Sample 3.1 Normal** | **Sample 3.2 Normal** | **Sample 3.1 Tumor** | **Sample 3.2 Tumor** |  |  |  |  |
| **ρ** | **ρ** | **ρ** | **ρ** |  |  |  |  |
| 1,2 | 1,2 | 1,2 | 1,2 |  |  |  |  |
| **0.99638** | **0.98952** | **0.99146** | **0.99556** |  |  |  |  |
| 2,3 | 2,3 | 2,3 | 2,3 |  |  |  |  |
| **0.99747** | **0.99183** | **0.99780** | **0.99547** |  |  |  |  |
| 1,3 | 1,3 | 1,3 | 1,3 |  |  |  |  |
| **0.99155** | **0.98392** | **0.99525** | **0.99790** |  |  |  |  |
